# Targeting Myc-driven stress vulnerability in mutant *KRAS* colorectal cancer

**DOI:** 10.1186/s43556-022-00070-7

**Published:** 2022-03-21

**Authors:** Hang Ruan, Brian J. Leibowitz, Yingpeng Peng, Lin Shen, Lujia Chen, Charlie Kuang, Robert E. Schoen, Xinghua Lu, Lin Zhang, Jian Yu

**Affiliations:** 1 UPMC Hillman Cancer Center Research Pavilion, Suite 2.26h, 5117 Centre Ave., Pittsburgh, PA 15213 USA; 2 Department of Pathology, University of Pittsburgh School of Medicine, Pittsburgh, PA 15213 USA; 3 Department of Pharmacology and Chemical Biology, University of Pittsburgh School of Medicine, Pittsburgh, PA 15213 USA; 4 Department of Oncology, Xiangya Hospital, Central South University, Changsha, Hunan 410008 P.R. China; 5 Department of Medical Informatics, University of Pittsburgh School of Medicine, Pittsburgh, PA 15232 USA; 6 Department of Medicine, University of Pittsburgh School of Medicine, Pittsburgh, PA 15213 USA; 7 Department of Epidemiology, University of Pittsburgh School of Public Health Pittsburgh, Pittsburgh, PA 15213 USA

**Keywords:** Mutant *KRAS*, Myc, eIF2α, Colorectal cancer, Everolimus, Bortezomib

## Abstract

Mutant *KRAS* is a key driver in colorectal cancer (CRC) and promotes Myc translation and Myc-dependent stress adaptation and proliferation. Here, we report that the combination of two FDA-approved drugs Bortezomib and Everolimus (RAD001) (BR) is highly efficacious against mutant *KRAS* CRC cells. Mechanistically, the combination, not single agent, rapidly depletes Myc protein, not mRNA, and leads to GCN2- and p-eIF2α-dependent cell death through the activation of extrinsic and intrinsic apoptotic pathways. Cell death is selectively induced in mutant *KRAS* CRC cells with elevated basal Myc and p-eIF2α and is characterized by CHOP induction and transcriptional signatures in proteotoxicity, oxidative stress, metabolic inhibition, and immune activation. BR-induced p-GCN2/p-eIF2α elevation and cell death are strongly attenuated by *MYC* knockdown and enhanced by *MYC* overexpression. The BR combination is efficacious against mutant *KRAS* patient derived organoids (PDO) and xenografts (PDX) by inducing p-eIF2α/CHOP and cell death. Interestingly, an elevated four-gene *(DDIT3, GADD45B, CRYBA4 and *HSPA1L) stress signature is linked to shortened overall survival in CRC patients. These data support that Myc-dependent stress adaptation drives the progression of mutant *KRAS* CRC and serves as a therapeutic vulnerability, which can be targeted using dual translational inhibitors.

**Supplementary Information:**

The online version contains supplementary material available at 10.1186/s43556-022-00070-7.

## Introduction

Colorectal cancer (CRC) is the third most common cancer worldwide, with an estimated 1.2 million new cases and over 600,000 death annually [1]. Genetic alternations in oncogenes and tumor suppressors cooperate with epigenetic alterations to drive colorectal carcinogenesis [2]. *KRAS* is the most frequently mutated oncogene in human cancer. A comprehensive up to date analysis revealed that ~ 19% of cancer patients harbor *RAS* mutations and over 85% are in *KRAS,* equivalent to ~ 3.4 million new cases per year worldwide [3]. Mutational activation of *KRAS* is an early event in CRC development and occurs in about 40–50% of cases, with hot spots in codons 12, 13, 61, and 146 [2, 4]. Mutant *KRAS* is correlated with poor prognosis in CRC and resistance to Epidermal Growth Factor Receptor (EGFR) antibodies, and immune checkpoint blocker such as anti-PD1 [4]. Unfortunately, attempts to directly target mutant *KRAS* have limited success so far in the clinic. While selective KRAS G12C and G13C inhibitors demonstrated some promise in lung cancer patients recently [5, 6], these mutations however are present in less than 5% of CRCs. Therefore, targeting *KRAS* mutated CRCs remains a challenge.

Deregulated mRNA translation and protein synthesis is a common node of oncogenesis, as specialized proteins are required to sustain cancer hallmarks such as increased proliferation, altered metabolism, metastasis, and resistance to cell death or immune attack [7–10]. Mutant KRAS or BRAF increases proliferation and translation of many targets including oncogenic ones such as Myc, Bcl-xL and MMPs. This is thought to be mediated largely through the dissociation of Eukaryotic Translation Initiation Factor 4E (eIF4E) from its inhibitor 4EBPs upon their phosphorylation by MAPK/ERK and PI3K/mTOR/AKT signaling [11]. Elevated protein synthesis and demand on quality control in cancer cells lead to increased phosphorylation of Eukaryotic Translation Initiation Factor 2 alpha (eIF2α) (S51, p-eIF2α), the core regulator of the “integrated stress response” (ISR) [12]. There are four known eIF2α kinases in mammals which are activated by distinct and overlapping stresses. Among them, general control nonderepressible 2 (GCN2) is the most ancient and activated by amino acid starvation. A modest increase in p-eIF2α inhibits cap-dependent translation, while facilitating the translation of stress-related transcription factors such as Activating Transcription Factor 4 (ATF4) and C/EBP Homologous Protein (CHOP, encoded by *DDIT3*) to promote adaptation and survival through widespread changes in transcription, translation, metabolism, and myriad effectors [12, 13]. Failure to adapt leads to prolonged p-eIF2α elevation and CHOP induction, and subsequent cell death through apoptotic mediators such as death receptor 5 (DR5), p53 Upregulated Modulator of Apoptosis (PUMA), and Phorbol-12-myristate-13-acetate-induced protein 1 (also called NOXA) [12, 13].

Myc is a master regulator of oncogenic growth and metabolism [14–17], in part through elevated p-eIF2α and p-eIF4E/4EBP1 that promotes stress adaptation and survival of cancer cells [18–20]. This Myc-translation axis was therefore suggested as an exploitable vulnerability particularly in metabolically active tumors with mutated or amplified *KRAS* [16, 21]. However, targeting a single Myc target [14, 15], metabolic pathway, or a single step in mRNA translation [7, 8], yielded little clinical efficacy in most solid tumors. For example, inhibitors against mRNA Cap binding, eIF4A helicase [7, 8], or p-eIF2α inducing agents such as proteasome (Bortezomib and Ixazomib) [12, 22] and HSP90 inhibitors [23, 24], lack potency or selectivity. On the other hand, many kinase inhibitors target translation indirectly by blocking the phosphorylation of eIF4E or 4EBP [7, 8]. These include allosteric mTOR inhibitors Everolimus (i.e., RAD001) and Temsirolimus, and EGFR antibodies or inhibitors. However, mutant *KRAS* and *BRAF* represents a major resistance mechanism to single agent in CRC preclinical models or patients [25–28] due to complex feedback activation of survival pathways [4, 29]. Dual inhibition of ERK/MAPK and PI3K/mTOR signaling generally produces unacceptable normal tissue toxicity [7, 8].

We reasoned that Myc-driven stress adaptation is a critical survival mechanism in mutant *KRAS* CRCs, and Myc translation might be an useful therapeutic target [30]. In this study, we focused on FDA-approved agents and discovered that the combination of Bortezomib and Everolimus synergistically and selectively kills mutant *KRAS* CRC cells at concentrations where single agents had little or no toxicity. The efficacy of this combination is validated using patient derived organoids (PDOs) and xenografts (PDXs). Mechanistically, we showed that mutant *KRAS*-dependent vulnerability is mediated through elevated Myc, and its ablation leads to GCN2/p-eIF2α -dependent cell death with profound trancriptional signatures of proteotoxcity, oxidative stress and metabolic suppression. Our study provides a potential way to improve the treatment of mutant *KRAS* CRCs by targeting the deregulated Myc-ISR axis.

## Results

### Bortezomib and Everolimus synergistically kill mutant *KRAS* CRC cells

**Fig. 1:**
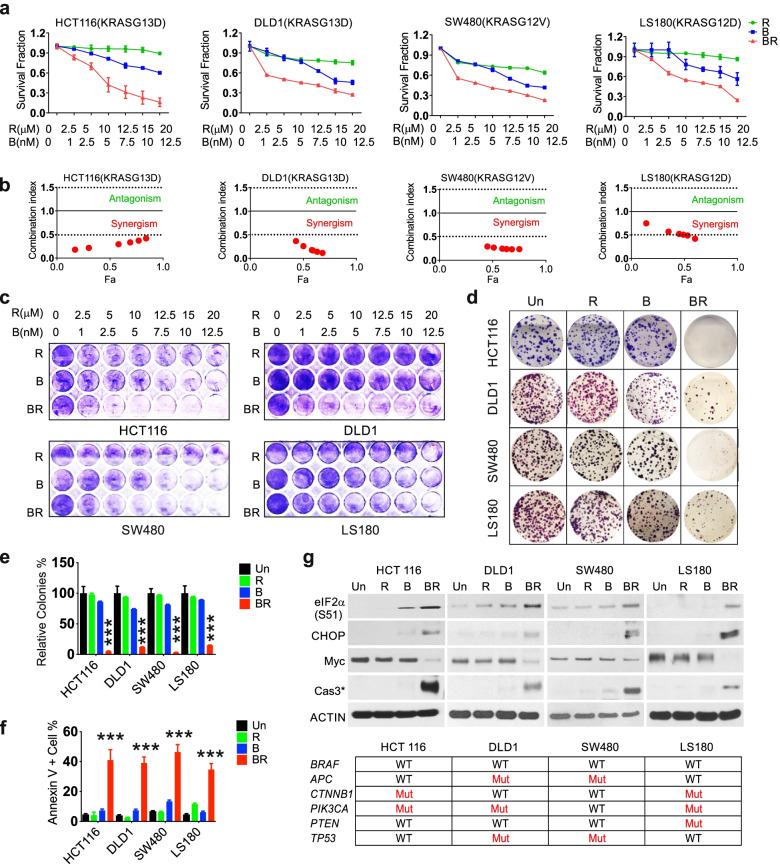
Bortezomib and Everolimus show synergistic antitumor effects in mutant *KRAS* CRC cells. Mutant *KRAS* HCT116, DLD1, SW480 and LS180 cells were treated with vehicle (untreated, Un), Bortezomib (B), Everolimus (R), or their combination (BR, 5 nM and 10 µM or specified ranges). **a** Cell growth at 48 h was assessed using MTS assay. **b** Calculated combination index (CI). The CI presents synergism (CI < 1), additive effect (CI = 1), and antagonism (CI > 1). Red area presents synergism, and green area presents antagonism. Fa represents fraction affected. **c** Attached cells were visualized by crystal violet at 48 h. (D) Colony formation assay. Cells were treated for 24 h and replated in drug free medium for 14 days before crystal violet staining. **e** The relative number of colonies as in (**d**) was normalized to untreated group (100%). **f** Apoptosis was quantitated by Annexin V + cells using flow cytometry. **g** Indicated proteins at 24 h was detected using western blotting. ACTIN is used as loading control. The status of major drivers is shown below. e, f, values are mean + s.d. (*n* = 3). ***p < 0.001 (Student’s *t*-test, two tailed). B, R *vs.* BR

To identify potential combination targeting mutant *KRAS* in CRC, we first performed a targeted drug screen using 11 translational inhibitors and HCT116 cells*.* These included pathway agents indirectly targeting p-4EBP1 or p-eIF4E, and ones targeting the cap binding complex (eIF4A, eIF4F assembly), or the 43S preinitiation complex (eIF2α). As expected, these agents showed massive differences in IC50 as single agent (over 20,000-fold, from low nM to sub mM). Cap analog 4Ei-1 was the least potent (at 500 µM or higher), while the p-eIF2α inducer bortezomib and eIF4A inhibitor were among the most potent (10–20 nM, Table S1). Direct or indirect kinase inhibitors had low to modest toxicity with IC50s ranging between 20–100 µM. The combination of Bortezomib (B) and Everolimus (R) (BR, hereafter) showed strong synergy in suppressing cell growth (Fig. 1a). Both are FDA-approved drugs and chosen for further study.

We validated the synergy of BR using three additional *KRAS* mutant CRC cells (DLD1, SW480 and LS180) and viability assay with combination index calculated (CI < 0.5) (Fig. 1a-b). The BR combination (B 5 nM and R 10 µM) strongly suppressed cell growth at 48 h (Fig. 1c), and long-term clonogenicity on day 14 with 24 h exposure. In contrast, either agent alone at the same doses showed little or no toxicity (Fig. 1d-e). The combination potently induced apoptosis as evidenced by increased Annexin V positive cells at 48 h (Fig. 1f and Fig. S1a) and cleaved caspase-3 at 24 h in all four lines (Fig. 1g). Compared to either agent, BR induced much higher levels of phosphorylation of eIF2α (S51) and CHOP, and a strong reduction in Myc protein in all four lines (Fig. 1g). These *KRAS* mutated lines are all WT for *BRAF* but otherwise vary in mutational status of *APC, CTNNB1 (β-catenin), PIK3CA, PTEN,* and *TP53* (Fig. 1g and Table S2). We further confirmed induction of prolonged ISR with elevated ATF4, GRP78/BIP and spliced *XBP1s* in HCT 116 cells by BR, not by single agent (Fig. S1b). BR and single agent reduced 4E-BP1 phosphorylation (S65/70) similarly, but had limited effect on 4E-BP1 (T37/46) or S6 phosphorylation (S235/236) (Fig. S1b). These results demonstrated that BR potently induces Myc ablation and stress-associated cell death in mutant *KRAS* cells.

### The BR combination induces p-eIF2α-dependent killing of mutant *KRAS* CRC cells

**Fig. 2:**
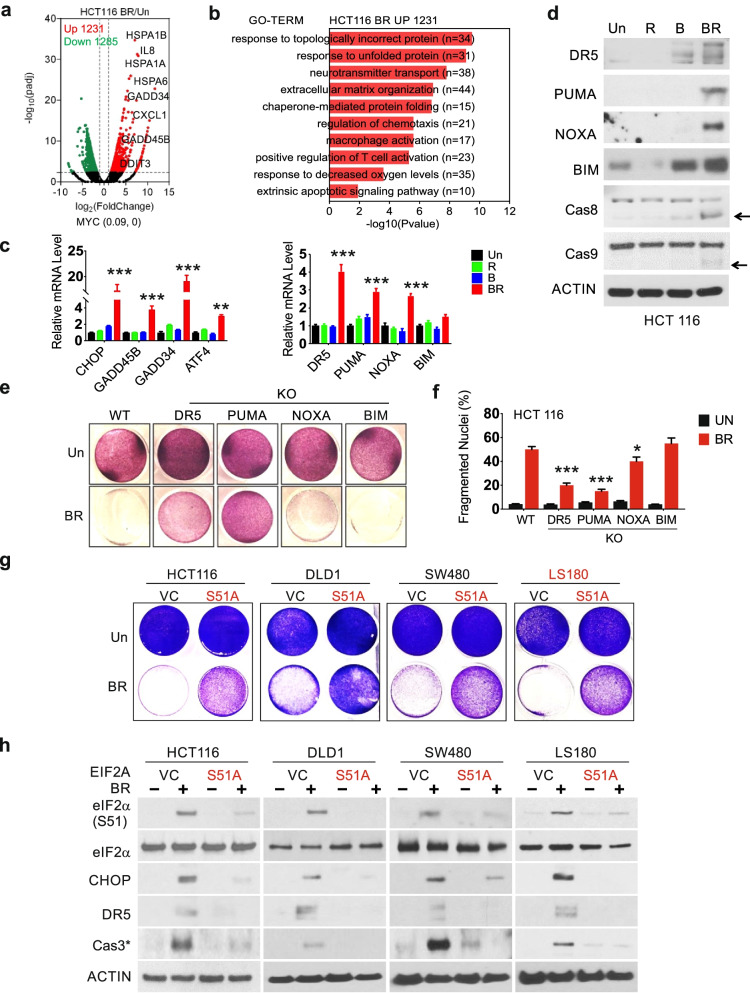
The BR combination induces p-eIF2α-dependent apoptosis of mutant *KRAS* CRC cells. The indicated cells were treated with vehicle (Un, Untreated), Bortezomib (B), Everolimus (R), or the combination (BR, 5 nM and 10 µM). **a** Differential genes induced by BR in HCT116 cells at 24 h visualized by volcano plot. Upregulated (red) or downregulated (green) genes (fold change ≥ 2, *p* < 0.005). Selected upregulated genes are shown. **b** Top 10 enriched non-overlapping pathways in upregulated genes (1231) identified by GO. **c** qRT-PCR analysis of the indicated markers at 24 h. **d** Western blotting of the indicated proteins at 24 h. Arrows indicate cleaved and active caspase. Actin was used as the loading control. **e** Attached cells at 48 h were visualized by crystal violet staining. **f** Apoptosis at 48 h was analyzed by nuclear fragmentation assay. **g** Cells were transfected with vector control (VC) or *EIF2AS51A* expression construct for 24 h, replated for 24 h, and treated by the BR combination for 48 h. Attached cells were visualized by crystal violet staining. **h** Western blotting of indicated proteins at 24 h. c and f, values are mean + s.d. (*n* = 3). *p < 0.05, **p < 0.01, ***p < 0.001 (Student’s *t*-test, two tailed). B, R *vs.* BR, or WT *vs.* KO

To further explore the mechanisms underlying BR-induced cell killing, we conducted RNA-Seq analysis on HCT116 cells. Differentially expressed genes (DEGs) included 1231 upregulated and 1285 downregulated by two-fold and more at 24 h (*p* ≤ 0.005) (Fig. 2a). Gene ontology (GO) analysis indicated that upregulated genes are highly enriched in pathways for proteostress and unfolded protein response (UPR), including multiple HSP family members and classical ISR markers *CHOP* and Growth Arrest and DNA Damage-Inducible Protein 45B and 34 (*GADD45B and GADD 34*) (Fig. 2a-b). Other highly upregulated pathways included extrinsic apoptosis, oxidative stress, and, surprisingly, immunity (Fig. 2b). The top 10 enriched pathways in downregulated genes were related to metabolism and cell cycle (Fig. S2a). Gene set enrichment analysis (GSEA) confirmed positive enrichment in misfolded proteins and leukocyte activation among others (Fig. S2b and data deposit).

qRT-PCR analysis confirmed the induction of the proximal ISR regulators *CHOP(DDIT3), GADD45B*, *GADD34,* and *ATF4,* as well as apoptotic effectors *DR5*, *PUMA, NOXA,* and *BIM* by the combination, but not single agents in HCT 116 cells (Fig. 2c). The induction of apoptotic proteins was also confirmed by western blotting with cleaved casapase-8 and -9 (Fig. 2d). Using isogenic HCT 116 cells deficient in one of these four apoptotic effectors [31, 32], we found that BR-induced cell killing is abrogated by *DR5* knockout (KO) or *PUMA* KO, but minimally affected by *NOXA* KO or *BIM* KO (Fig. 2e-f).

Prolonged p-eIF2α elevation and CHOP induction leads to cell death [12]. p-eIF2α can be inhibited by the overexpression of a phosphorylation defective and dominant negative mutant allele (serine 51 to alanine, herein referred to as S51A) [10, 28]. The expression of *eIF2AS51A* significantly blocked BR-induced cell loss, with marked reduction in p-eIF2α, CHOP, DR5, and cleaved caspase-3 (Fig. 2g-h), and expression of ISR targets *CHOP, GADD45B, GADD34*, *ATF4*, and apoptotic effectors (*DR5, PUMA, NOXA *and* BIM*) (Fig. S2c). Inhibition of apoptosis was confirmed by nuclear fragmentation assay and flow cytometry (Fig. S2d-f). These data demonstrate that sustained p-eIF2α leads to the killing of mutant *KRAS* CRC cells through the activation of intrinsic and extrinsic apoptotic pathways upon BR treatment.

### The BR combination triggers Myc-dependent activation of GCN2

**Fig. 3:**
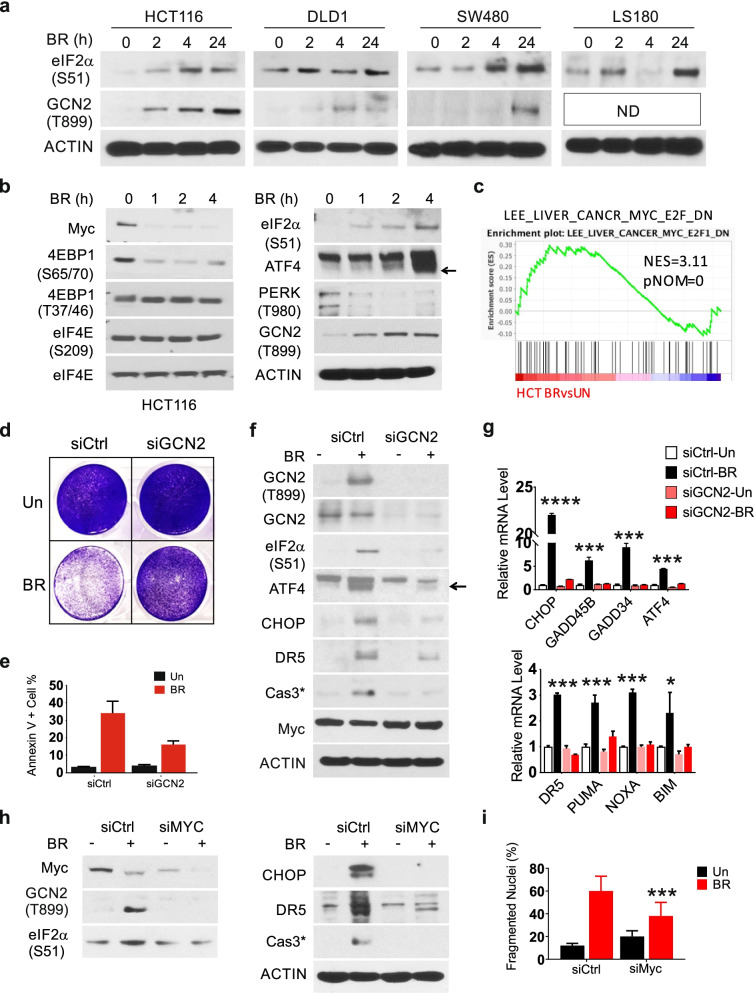
The BR combination triggers Myc- and GCN2-dependent apoptosis. Indicated cells were treated with vehicle (Un), or the Bortezomib (B), and Everolimus (R) combination (BR, 5 nM and 10 µM). **a** Western blotting of indicated proteins at 0, 2, 4 and 24 h. ND, not detected. **b** Western blotting of indicated proteins at 0, 1, 2 and 4 h. (**c)**. GSEA of differential genes in the indicated pair (C2 dataset). The indicated gene set is shown with NES (normalized enrichment score) and corresponding *p-*value. **d** Cells were transfected with either scramble or GCN2 siRNA for 24 h, replated for 24 h and treated with BR for 48 h. Attached cells were visualized by crystal violet staining. **e** Quantification of Annexin V + cells. **f** Western blotting of indicated proteins, and (**g**) qRT-PCR analysis of indicated genes at 24 h. The arrow indicates the specific lower band. **h** Cells were transfected with either scrambled (Ctrl) or MYC siRNA and treated with BR as in d, analyzed by western blotting at 24 h and **i** apoptosis at 48 h. g, i, values are mean + s.d. (*n* = 3). *p < 0.05, ***p < 0.001, ****p < 0.0001 (Student’s *t*-test, two tailed). Control (Ctrl) *vs.* siRNA

The strong metabolic suppression by BR observed at 24 h (Fig. S2a) prompted us to examine the role of GCN2, a classical metabolic stress sensor and eIF2α kinase [13]. Elevation of p-eIF2α was observed at 2–4 h in HCT 116, DLD1, SW480, and LS180 cells (Fig. 3a), in parallel with elevated p-GCN2 in 3 of 4 lines. Despite decreased Myc protein (Fig. 1g), little or no change in *MYC* mRNA was detected at 24 h in any line (Fig. S3a). Interestingly, loss of Myc and 4EBP1 (S65/70, not T37/46) was rapid and near complete at one hour, when p-GCN2, p-eIF2α and ATF4 just began to rise, continuing to 4 and 24 h in HCT 116 cells (Fig. 3b, Fig. S3b). We detected no change in total eIF4E or p-eIF4E (S209) and decreased p-PERK, the UPR sensor (Fig. 3b). The levels of CHOP and death effectors such as DR5 and cleaved caspase-3 began to rise only at or after 12 h (Fig. S3b). Consistent with Myc loss, significant enrichment of Myc down-regulated targets was evident at 24 h (Fig. 3c).

We further examined the role of GCN2 and Myc in BR-induced cell death. *GCN2* siRNA markedly reduced BR-induced growth inhibition and apoptosis (Fig. 3d-e, Fig, S3c), as well as the levels of p-eIF2α, ATF4, CHOP, caspase-3 cleavage, or ISR targets, with a minor effect on Myc loss (Fig. 3f-g). *MYC* siRNA strongly reduced BR-induced cell death, p-GCN2/p-eIF2α/CHOP and caspase-3 cleavage (Fig. 3h-i). These results suggest that acute Myc loss in mutant *KRAS* CRC cells impairs stress adaptation and leads to GCN2/p-eIF2α-dependent metabolic crisis and cell death.

### The BR combination promotes mutant *KRAS*-Myc selective proteotoxicity

**Fig. 4:**
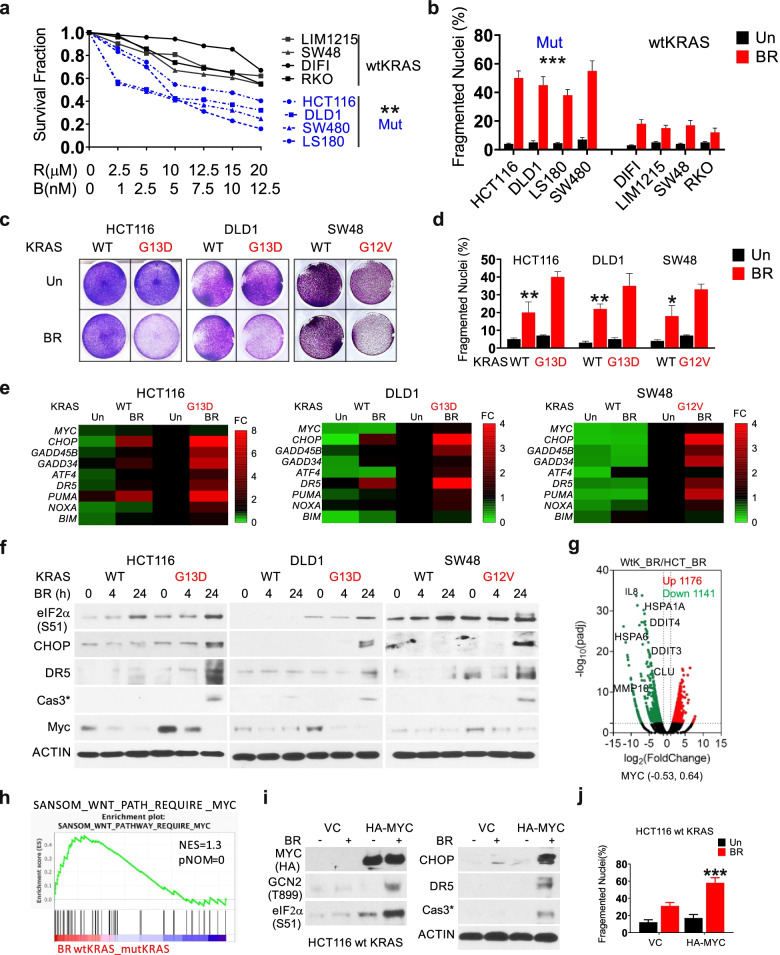
The BR combination induces mutant *KRAS*-selective stress hyperactivation and cell death. CRC cells with either WT or mutant *KRAS* were treated with vehicle (Un), or the Bortezomib (B) and Everolimus (R) combination (BR, 5 nM and 10 µM, or as specified). **a** Dose response of 8 cell lines at 48 h was assessed by MTS assay. Dotted (blue) or solid lines represented cells with MUT or wtKRAS. **b** Apoptosis at 48 h was analyzed by nuclear fragmentation assay. **c** Attached cells at 48 h were visualized by crystal violet staining. **d** Apoptosis at 48 h was analyzed by nuclear fragmentation assay. **e** qRT-PCR analysis of indicated genes at 24 h visualized by heatmap. The expression was normalized to untreated isogenic mutant *KRAS* cells (1). **f** Western blotting of indicated proteins at 0, 4 and 24 h. **g** DEGs (FC ≥ 2, *p* < 0.005) in BR-treated WT *vs.* mut *KRAS* HCT 116 cells at 24 h visualized by volcano plot. Selected down-regulated genes (green) in WT *KRAS* cells are shown. **h** GSEA of differential genes in the indicate pair (C2 dataset). The indicated gene set is shown with NES and corresponding *p*-value. **i** Cells were transfected with either empty vector (VC) or HA-MYC plasmid for 24 h, replated for 24 h, and treated with BR. Western blotting of indicated proteins at 24 h, and **j** apoptosis at 48 h analyzed by nuclear fragmentation assay. a, b, d, j, values are mean + s.d. (*n* = 3). *p < 0.05, **p < 0.01, ***p < 0.001, ****p < 0.0001 (Student’s *t*-test, two tailed). WT *vs.* mut *KRAS* BR group or cell line, or VC *vs*. HA-MYC

We further determined if elevated Myc in mutant *KRAS* CRC cells [30] is the target of BR. Compared to mutant *KRAS* CRC cells (n = 4), WT *KRAS* CRC cells (n = 4) showed higher IC50, reduced apoptosis, and lower induction of stress (p-eIF2α, CHOP, GADD45B), DR5, and cleaved caspase-3 (Fig. 4a-b, Fig. S4a-b). To minimize the influence of genetic background, we took advantage of isogenic mutant and WT *KRAS* CRC cell pairs previously established [33]. We confirmed higher BR sensitivity in mutant *KRAS* isogenic cells (HCT116, DLD1 and SW48) by growth suppression and apoptosis induction (Fig. 4c-d). qRT-PCR indicated induction of some ISR regulators and apoptotic effector genes (8) by BR in WT *KRAS* isogenic cells, albeit at much reduced levels compared to those in mutant *KRAS* counterparts (Fig. 4e). Myc protein, not *MYC* mRNA, was effectively ablated by BR within all lines (Fig. 4e-f). Isogenic WT *KRAS* cells showed lower basal Myc and p-eIF2α, and lacked the induction in CHOP, apoptotic targets, or cleaved caspase-3 at 24 h (Fig. 4f).

We then used RNA-seq to compare global transcriptional response of isogenic WT and mutant *KRAS* HCT 116 cells to BR treatment. Consistent with the lack of acute stress or death, WT *KRAS* cells displayed a drastically reduced global transcriptomic response (Fig. S4c). BR-treated WT *KRAS* cells showed profound decrease of pathways in proteostress, intrinsic and extrinsic apoptosis, and HIF1α and immunity (-Log10 (pvalue) from 15–5) (Fig. 4g, Fig. S4d), and increase of pathways in development, cell cycle, metabolism, and chromosome segregation (Fig. S4e). Interestingly, top BR differential genes appeared to be regulated at opposite directions in this isogenic pair (Fig. S4f-g). Despite little or no change in *MYC* mRNA, GSEA showed negative enrichment of Wnt-Myc in BR-treated mutant *KRAS* cells (Fig. 4g-h). Overexpression of *MYC* in WT *KRAS* cells increased p-GCN2/p-eIF2α/CHOP, and DR5 and cleaved caspase-3 at 24 h and cell death at 48 h upon BR treatment, with (Fig. 4i-j). Together with results from *MYC* siRNA, these data demonstrate a fundamental role of Myc in regulating metabolic and transcriptomic response in mutant *KRAS* CRC cells.

### The BR combination is effective against mutant *KRAS* CRC PDOs

**Fig. 5:**
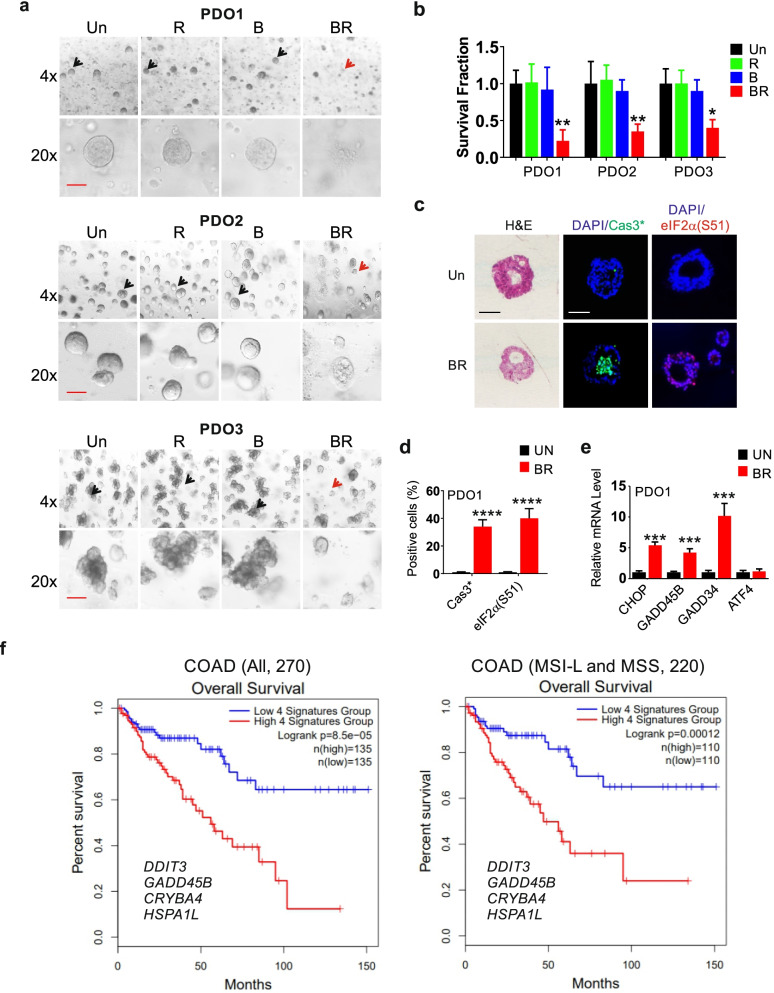
The BR combination induces ISR hyperactivation and killing of PDOs. CRC PDOs were treated with vehicle (Un), Bortezomib (B), Everolimus (R), or their combination (BR, 7.5 nM and 15 µM). **a** Representative images of PDOs at 48 h. Live and dead organoids are indicated by black and red arrows, respectively. Scale bar = 100 µM. **b** The growth of PDOs treated as in (A) was quantified by 3D cell viability assay**. c** Representative images of H&E, and cleaved caspase 3 and p-eIF2α IF at 24 h in Paraffin-embedded PDO1 sections. Scale bar = 50 µM. **d** Quantitation of cleaved-caspase 3 and p-eIF2α IF in **c**. A minimum of 30 organoids were analyzed for each condition. **e** qRT-PCR analysis at 24 h with values normalized to un (1). b, d, e, values are mean + s.d. (*n* = 3, 8-well pool). *p < 0.05, **p < 0.01, ***p < 0.001, ****p < 0.0001 (Student’s *t-*test, two tailed). Un *vs.* BR. **f** Correlation of four stress-gene signature and OS in the COAD (ALL) or the MSL-L (*n* = 54) and MSS (*n* = 182) subgroups. Log-rank test

Patient derived organoids (PDOs) retain the architecture, genotype, and phenotype of the patient’s primary tumor and provide a rapid in vitro model for drug testing [34]. We then tested the efficacy of BR using mutant *KRAS* CRC PDOs. These PDOs had two distinct morphologies as cysts or clusters [35], and did not differ significantly in growth (Fig. S5a). PDOs were significantly more sensitive to the BR combination, compared to single agent (Fig. 5a-b). The BR treatment induced marked apoptosis (cleaved-caspase-3) in the center of PDO, along with highly elevated p-eIF2α and expression of ISR effectors at 24 h (Fig. 5c-e, Fig. S5b).

Our data support that mutant *KRAS* is linked to elevated Myc and ISR in CRC. Mutant *KRAS* was not significantly correlated with microsatellite instability (MSI) status, and correlated with shortened median overall survival (OS) in CRC patients (59.93 *vs.* 78.73 months) (n = 1965, cBioportal) (Fig. S5c-d). However, ISR effectors are numerous and regulated by oncogenic drivers other than mutant *KRAS* or Myc [10, 28]. We therefore examined potential prognostic values of ISR targets using a web-based database GEPIA2 (Gene Expression Profiling Interactive Analysis) [36]. Higher expression of *CHOP(DDIT3)*, *GADD45B*, *CRYBA4*, and *HSPA1L* was associated with shortened overall survival (OS) in TCGA (The Cancer Genome Atlas) COAD cohort (n = 270) (Fig. S5e). *CHOP(DDIT3)*, *GADD45B*, *CRYBA4* were induced by BR in mutant *KRAS* CRC cells (Figs. 2 and 4). Remarkably, the 4-gene signature predicts OS better than any single gene (Log-rank *p* = 7.1e-05, HR (Hazard ratio) = 2.7, *p*(HR) = 0.001384) in this cohort (n = 270), as well as in the MSI-L (n = 52) and microsatellite stable (MSS) (n = 184) subsets (Log-rank *p* = 0.00012, HR = 2.9, *p*(HR) = 0.00023) (Fig. 5f, Fig.S5e).

### The BR combination shows potent efficacy in mutant *KRAS* MSS CRC PDXs

**Fig. 6:**
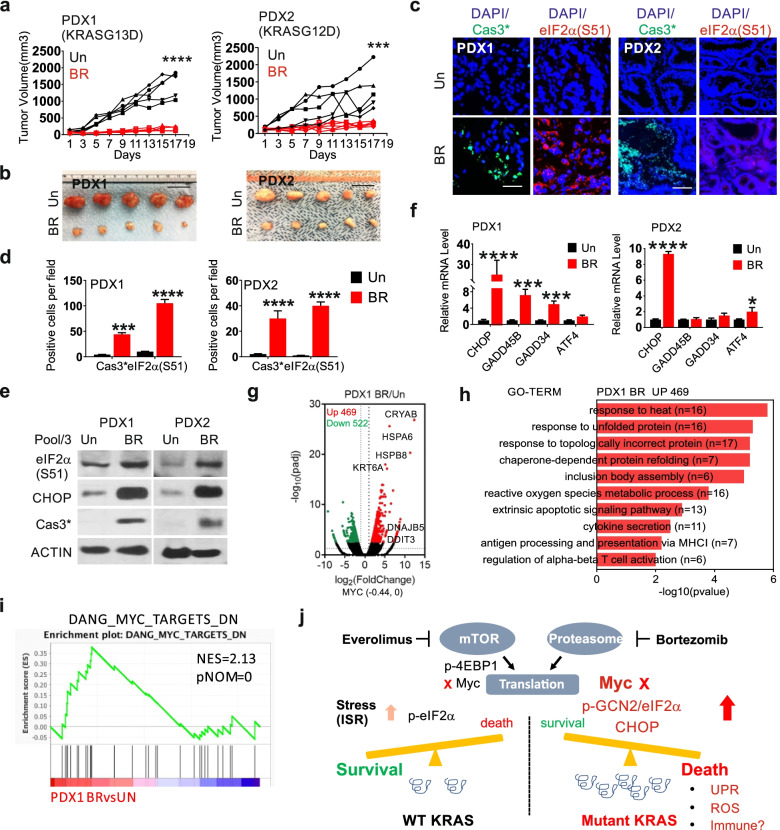
The BR combination kills mutant *KRAS* MSS CRC PDXs. NSG were randomized into control and treatment groups when average PDX volume reached around 100 mm3. Mice were treated with vehicle (Un), or the combination of Everolimus (oral gavage, 10 mg/kg) and Bortezomib (i.p., 0.5 mg/kg) (BR) every other day starting day 1. Tumors were harvested for analysis. **a** Individual tumor volume was calculated and plotted (*n* = 5) with **b** representative images on day 17. Scale Bar = 2 cm. **c** Representative cleaved caspase 3 and p-eIF2α IF with DAPI counterstain (blue) on day 4. Scale Bar = 100 µM. **d** Quantification of cleaved-caspase 3 and p-eIF2α IF as (**c**) in 3 randomly chosen 400X fields. **e** Western blotting of indicated proteins on day 4. *N* = 3 (pooled). **f** qRT-PCR of indicated makers on day 4. The values were normalized to Un (1). *N* = 3 (pooled mRNA). **g** BR DEGs in PDX1 on day 4 visualized by volcano plot. Upregulated (red) or down regulated (green) genes (fold ≥ 2, *p* < 0.005). Selected upregulated genes are shown. **h** Top 10 enriched non-overlapping pathways identified by GO in upregulated genes (469). **i**. GSEA of differential genes in PDX1 (C2 dataset). The indicated gene set is shown with NES and corresponding *p*-value. a, d, f, values are mean + s.d. (*n* = 3 or as specified). *p < 0.05, **p < 0.01, ***p < 0.001, ****p < 0.0001 (Student’s *t*-test, two tailed). Vehicle (Un) *vs.* BR. **j** Working model. Targeting Myc-driven stress in *KRAS* mutated CRCs. Compared to WT *KRAS* CRCs, mutant *KRAS* CRCs show elevated basal Myc and metabolic stress. Acute ablation of Myc protein by the BR combination impairs their adaptation, leading to sustained ISR (p-GCN2/p-eIF2α/CHOP) and cell death associated with UPR, reactive oxygen species (ROS) production, and immune activation

The above data suggest that Myc-driven stress adaptation might be a selective target in aggressive CRCs. Patient derived xenograft (PDX) models preserved tumor histology and heterogeneity [37], and were used to test the efficacy of the BR combination. We selected two PDXs (mutant *KRAS* and MSS). PDX1 (KRASG13D) and PDO1 were originated from the same tumor. PDX2 (KRASG12D) is therapy resistant and from a metastatic lesion of deceased patient after multiple lines of chemo- and targeted therapies (FOLFIRI, FOLFOX, 5-FU, bevacizumab, onalespib, indimitecan, selumetinib and MK-2206). PDX1 and PDX2 were highly responsive to the BR treatment (Fig. 6a-b, Fig. S6a), showing massive loss of proliferation (Ki67 index) and reduction in cellularity upon BR treatment (Fig. S6b-c). The BR treatment was well tolerated and induced a slight and transient weight loss compared to the control group, which recovered by day 9 (Fig. S6d).

To monitor drug-induced acute response *in vivo*, we first analyzed several ISR and cell death markers in PDXs on day 4, which is 24 h after the second BR treatment. BR group showed significant increases in p-eIF2α, CHOP and cleaved caspase-3 by staining and western blotting (Fig. 6c-e). qRT-PCR confirmed the induction of ISR effectors (Fig. 6f, Fig. S6e), and a similar pattern between PDO1 and PDX1. We used RNA-Seq to assess BR-induced global transcriptomic changes in PDX1, and identified 469 upregulated and 522 downregulated genes (filtered for human reads,twofold or more, *p* ≤ 0.005). Upregulated genes were highly enriched in proteo- and oxidative stress, apoptosis, and immunity (Fig. 6g-h), while downregulated genes were highly enriched in metabolism such as oxidative phosphorylation and nucleotide and ATP biosynthesis (Fig. S6f). Upregulated genes shared by HCT 116 and PDX1 were highly enriched in proteostress and extrinsic apoptotic pathway (Fig. S6g-h). Like cell line studies, BR treatments significantly enriched Myc down-regulated targets (Fig. 6i), with little effect on *MYC* mRNA (Fig. 6g). These data collectively support that the BR combination targets Myc-dependent stress adaptation in mutant *KRAS* CRCs to promote ISR-dependent metabolic crisis and cell death (Fig. 6j).

## Discussion

Targeting mutant *KRAS* is a major clinical challenge and the “holy grail” in cancer therapy. Mutant *KRAS* is linked to poor prognosis in CRC, and promotes resistance to EGFR antibodies [4] and anti-PD-1 [38]. Resistance is in part mediated through feedback activition of adaptive responses to avoid drug or immune-mediated cell killing [4, 39]. Here, we report a highly effective combination of two FDA-approved drugs, Bortezomib and Everolimus, against mutant *KRAS* CRC cells, PDOs, and PDXs. Mechanistically, the BR combination, but not single agents, ablates high Myc levels in mutant *KRAS* CRC, leading to unresolvable proteostress and cell death with a transcrptomic signature charaterized by proteotoxicity, oxidative stress, metabolic suppression, and immune activation (Fig. 6j). The vulerability to this drug combination is seletive to the mutant *KRAS*-Myc axis as demonstrated using isogenic and nonisogenic CRC cells. Our study therefore provides a potentially new therapeutic strategy to target Myc in mutant *KRAS* CRCs using FDA-approved drugs.

KRAS is a membrane-bound GTPase that cycles between GTP-bound active and GDP-bound inactive forms. Most oncognic mutations affect this on–off switch and lock the protein in the active form to drive cell proliferation, apoptosis resistance, and metastasis [4]. *KRAS* mutations are biochemcially distinct and appear to influence CRC patient outcomes [5], making it challening to develope allele spcific RAS inhibitors. Despite some encouraging data on G12C and G13C inhibitors in lung cancer [5, 6], G12D and G12V mutations are the most prevalent *KRAS* mutations in CRC [3] associated with worse overall survival [40]. Indirectly targeting mutant KRAS also has not had much succes [4, 29]. Our data suggest elevated Myc and protesostress as a druggable vunerability across mutant *KRAS* CRC cell lines, PDXs and PDOs using BR combination. Elevated proteostress is also reported in cancers with amplified *KRAS* [21] or MSI-high [41]. *KRAS* mutations are prevalent in pancreatic cancer (90%), lung cancer (20–30%), and endometrial cancer (18%) [3]. It is tempting to sepculate that this combination might be effective in other epithelial cancers with mutant *KRAS*.

Myc is a master regulator of oncogenic growth through extensive transcriptional and translational networks, and cooperates with a variety of cofactors [16, 42, 43]. Myc promotes stress adaptation and survival of cancer cells by increased autophagy [44], preservation of bioenergetics [45] through transient inhibition of RNA pol II-mediated transcription [46] or protein synthesis through GCN2/p-eIF2α-dependent negative feedback [28]. Myc translation is highly regulated and enhanced by mutant KRAS [30, 47]. However, this Myc-translation feed-forward loop is notoriously difficult to break [14, 15]. Our data support that Myc ablation is likely required to disrupt these protective mechanisms in mutant *KRAS* cells ‘addicted” to Myc (Fig. 6j). Consistent with this model, BR, but not single agents, rapidly ablates Myc protein, not mRNA, leading to sustained induction of p-GCN/p-eIF2α/CHOP. *MYC* siRNA decreased sensitivity while *MYC* over-expression increased sensitivity through the p-GCN/p-eIF2α/CHOP axis in mutant and WT *KRAS* isogenic cells, respectively. The combination is therefore necessary to push mutant *KRAS* CRC cells out of Myc-dependent adaptive “Goldilocks Zone” [8] and into metabolic crisis and cell death. However, the role of other eIF2α kinases in cell death cannot be ruled out due to their well-documented crosstalk and shared downstream targets [7, 12, 13, 48].

CRCs are heterogeous and can be classified into several molecular groups based on gene expression [49]. The majority of CRC are MSI-L and MSS, associated with a high Wnt/Myc signature, and do not respond to immune checkpoint inhibitors such as anti-PD-1 [50, 51]. Overwhelming evidence supports the cooperation of mutant *KRAS* and Myc in metabolic reprogramming and therapeutic resistance through the tumor microenvironment [18–20, 52]. Here, we found that the 4-gene ISR signature (*DDIT3, GADD45B, *CRYBA4, and HSPA1L) is strongly associated with poor prognosis in CRC patients, but also with sensitivity to BR in vitro and *in vivo.* Our model (Fig. 6j) helps explain this paradox. As mutant *KRAS* cells become dependent on elevated Myc and ISR (p-GCN2/p-eIF2α) for metabolic adaptation and immune suppression, acute Myc ablation breaks this state by inducing even higher and sustained ISR and cell death (Fig. 6j). Mutant KRAS-Myc strongly increases the range of transcriptional response and level of p-eIF2α to BR treatment, but it remains likely that additional BR targets are involved given the large number of Myc cofactors [16, 42, 43]. Elevated p-eIF2α is a suggested marker of immunogenic cell death (ICD) [53, 54]*.* The use of syngeneic models can help better understand drug-induced synthetic lethality for diffucult targets [55] through the tumor microenvironment (TME). The challenge however remains to develop mechanism-based clinical biomarkers distinct from activated oncogenes. Our data support that PDO and PDX might be useful in this regard, as those from the same patient showed similar stress-related pathway gene expression changes.

In summary, our study demonstrates a critical role of Myc-mediated stress adaptation in the surival of mutant *KRAS* CRC. The potent efficacy of the combination of FDA-approved mTOR and proteasome inhibitors is mediated through Myc ablation and induction of p-GCN2/p-eIF2α-dependent cell killing. With multiple FDA-approved agents in each class, the prevelance of *KRAS* mutations, and mechanistic biomarkers, it would be interesting to design clinical trials to evaluate potential benefit of BR or similar combinations in CRC patients.

## Material and methods

### Approval and protocols

All methods were performed in accordance with the relevant guidelines and regulations. The protocols for the use of recombinant DNA and animals included IBC201700136 and IACUC# 17071072. The protocol of establishing PDO/PDX includes REN11110076/IRB0411047.

### Cell culture, treatment, and transfection

The human colorectal cancer cell lines, including HCT116, DLD1, SW480, LS180, LIM1215, SW48, DIFI and RKO were obtained from the American Type Culture Collection (Manassas, VA, USA) ATCC. Isogenic *KRAS* pairs HCT116 (WT, G13D), DLD1 (WT, G13D) and SW48 (WT/G12V) cell lines were obtained from Bert Vogelstein at Johns Hopkins University [33]. *PUMA* KO [31], *DR5* KO, *NOXA* KO, *BIM* KO [32, 56] HCT 116 cells were generated in the lab. Information on major drivers or isogenic cell lines are found in Supplementary Materials (Table S1). Cell lines were regularly monitored for absence of Mycoplasma, approximately every 6 months. Any cell line is used for less than 2 months (10 or fewer passages) in culture upon thawing from LN tank. All cell lines were cultured in McCoy’s 5A modified medium (Invitrogen, Carlsbad, CA, Cat#16,600–082) supplemented with 10% defined fetal bovine serum (Hyclone, Logan, UT, Cat #SH3007103), 100 units/mL penicillin, and 100 µg/mL streptomycin (Invitrogen, Carlsbad, CA, Cat#15,140,148) unless noted otherwise. More details on drug treatment and transfection are found in the supplemental material.

Cell viability was performed using the MTT assay kit (Promega, Madison, WI, #G3580) as described [30]. In brief, cells were seeded in 96-well plates at a density of 1 × 10^**4**^ cells/well and treated with different agents for 48 h before the analysis. Combination index(CI) and fraction affected(Fa) values were calculated using Compusin software (https://www.combosyn.com).

Crystal violet staining. Following various treatment, attached cells or clones were stained and with crystal violet (Sigma, St. Louis, MO, Cat# C0775) (3.7% Paraformaldehyde, 0.05% crystal violet in distilled water and filtered at 0.45 um before use) (2). For colony formation assays, equal numbers of cells were subjected to various treatments and plated into 12-well plates at different dilutions. Colonies were visualized by crystal violet staining 14 days after plating. Each assay was conducted in triplicate and repeated three times.

### Apoptosis assays

Cell death and apoptosis was analyzed by nuclear staining with cells harvested from 12-well plates and Hoechst 33,258 (Invitrogen, Cat# 40,045), and Annexin V/propidium iodide (PI) followed by flow cytometry as described [57]. Experiments were repeated on two or more occasions (different days) with similar results. Adherent and floating cells were harvested, stained with Hoechst 33,258 (Invitrogen), and analyzed for apoptosis by nuclear staining and counting cells with condensed and fragmented nuclei. At least 300 cells were analyzed for each treatment. Annexin V/propidium iodide (PI) staining was performed. Flow cytometry plots and quantitation were based on the analysis of 20,000 cells for each condition. Results from one representative experiment are shown with fraction (%) of indicated population.

### Western blotting and quantitative Reverse transcription PCR (qRT-PCR)

Western blotting was performed as previously described [26, 30]. Details on antibodies are found in the Supplementary Materials (Table S4). Total RNA was isolated from cells or tissues using Mini RNA Isolation II Kit (Zymo Research, Orange, CA, Cat# R1054) according to the manufacturer’s protocol. One μg of total RNA was used to generate cDNA using SuperScript III reverse transcriptase (Invitrogen, Carlsbad, CA, Cat# 18,064–014). Details on primers are found in the Supplementary Materials (Table S5). For organoids, pooling of 8 or more wells for each condition is necessary to prepare enough lysates or total RNA. For tumors, lysates were pooled from 3 randomly chosen tumors in each group. cDNA was synthesized from RNA pooled from 3 randomly chosen tumors in each group.

### Patient derived CRC organoids (PDOs)

PDOs were established using surgically resected de-identified CRC tissues from the Biospecimen Core (PBC) at University of Pittsburgh with tissue collection under informed consent and usage approved by the Institutional Review Board at the University of Pittsburgh. CRC organoids were cultured in Matrigel (Corning) incubated with advanced DMEM/F12 (Invitrogen) medium with supplements including 50% (v/v) L-WRN-conditioned medium containing Wnt3a, R-spondin, and Noggin prepared as described [30, 58]. More details on medium, passage and treatment are found in supplemental materials.

### Patient derived CRC xenografts (PDX)

Animal experiments were approved by the University of Pittsburgh Institutional Animal Care and Use Committee. All methods were performed in accordance with the relevant guidelines and regulations. Patient-derived xenograft (PDX) tumors were established and propagated in 5–6-week-old female NOD.Cg-Prkdcscid Il2rgtm1Wjl/Saju (NSG) mice (Jackson Laboratory, Bar Harbor, ME) as described (4, 10) using samples collected with IRB approval and obtained from the NCI.

Tumor-bearing mice were randomized into untreated and treated groups. Mice were treated with BR Injection every other day. B was given by intraperitoneal (IP) injection at 0.5 mg/kg, R was given by oral gavage (OG) at 10 mg/kg. Tumor growth was monitored by calipers, and tumor volumes were calculated according to the formula 1/2 × length × width^2^. Ethical endpoint was defined as a time point when a tumor reached 2 cm or more in any dimension. Tumor tissues were analyzed for histology, staining, protein, and mRNA expression. Selected tumors were pooled to prepare protein or RNA which was then used for western blotting, RT-PCR and RNA-seq. More Details on PDX establishment and treatment and analysis are found in supplemental materials.

### RNA sequencing (RNA-Seq)

Total RNA was prepared from cells and tissues using TRIzol RNA Isolation Reagents (Sigma, St. Louis, MO, Cat# 15,596,026) according to manufacturer’s instructions. Library construction, RNA sequencing (RNA-seq), and data analysis were performed by Novogene using the IIIumina HiSeq platform. Sample quality was assessed by HTSeq v0.6.1 to the count the read numbers mapped of each gene. FPKM (fragments per kilobase of transcript per million mapped reads) of each gene was calculated based on the length of a gene and read counts mapped to this gene. For PDX samples, Flow B was used to filter and map human reads with Fragments Per Kilobase Per Million (FPKM) calculated based on all mapped reads. For samples without biological replicates, readcount was adjusted by TMM, then differential expression significant analysis was performed by using the edgeR package, while the significant criterion are both p value < 0.005 and |log2(Fold Change)|> 1 (i.e., twofold).

### Bioinformatics

More details on differential expression analysis and visualization such as Volcano plots, Gene Ontology (GO), Gene Set Enrichment Analysis (GSEA) and Venn diagram are found in supplemental materials.

### Data deposit

Analyzed RNA-seq data on differential gene expression, including raw readcount and normalized abundance of all called genes in paired samples (DEG, no cutoff), and differentially expressed genes (DEG_all, including both ups and downs, with indicated cutoff), additional gene list (s) and analyses(GSEA) are deposited at DRYAD. This dataset has been assigned a unique identifier or DOI (https://doi.org/10.5061/dryad.sf7m0cg6h) for free public access upon the publication of manuscript.

### Statistical analysis

Statistical analyses were carried out using GraphPad Prism software (VIII, GraphPad Software, Inc., La Jolla, CA). Comparisons between two groups were made by two-tailed, unpaired *t* test. Differences were considered significant if the probability of the difference occurring by chance was less than 5 in 100 (*p* < 0.05). The means ± one standard deviation (s.d.) were displayed in the figures. Sample size was determined using a combination of published work and power calculations.

## Supplementary Information

**Additional file 1: Supplementary Table S1. **IC50s of translation-targeted agents in HCT 116 cells. Supplementary Table S2. Cell line information. Supplementary Table S3. chemicals, siRNA, and other key reagents. Supplementary Table S4. Antibodies used in the study.** Supplementary Table S5. **qRT-PCR primers used (Human). Supplementary Figure S1. The BR combination induces apoptosis in mutant KRAS CRC cells. **Supplementary Figure S2. **The BR combination induces metabolic suppression and prolonged ISR. Supplementary Figure S3. BR induces ISR hyperactivation without affecting MYC mRNA.** Supplementary Figure S4. **BR induces mutant KRAS-selective ISR hyperactivation.** Supplementary Figure S5. **BR induces ISR-associated killing of CRC PDOs. **Supplementary Figure S6. **BR induces ISR hyperactivation and metabolic crisis in mutant KRAS CRC PDXs.

## Data Availability

All data needed to evaluate the conclusions in the paper are present in the paper and/or the Supplementary Materials. RNA-seq data and associated analyses have been deposited at DRYAD with a unique identifier or DOI (10.5061/dryad.sf7m0cg6h).

## Abbreviations

CRC: Colorectal cancer
EGFR: Epidermal Growth Factor Receptor
ISR: The Integrated Stress Response
eIF2α: Eukaryotic Translation Initiation Factor 2 alpha
eIF4E: Eukaryotic Translation Initiation Factor 4E
GCN2: General Control Nonderepressible 2
ATF4: Activating Transcription Factor 4
CHOP: C/EBP Homologous Protein
PERK: Protein Kinase RNA-Like ER Kinase
UPR: Unfolded Protein Response
ROS: Reactive Oxygen Species
GADD45B: Growth Arrest and DNA Damage-Inducible Protein 45 Beta
GADD34: Growth Arrest And DNA Damage-Inducible Protein 34
DR5: Death receptor 5
PUMA: P53 Upregulated Modulator of Apoptosis (encoded by *BBC3*)
PMAIP1, or NOXA: Phorbol-12-myristate-13-acetate-induced protein 1
BIM: Bcl-2-like protein 11
MSI: Microsatellite instability
MSS: Microsatellite stable (MSS)
TCGA: The Cancer Genome Atlas
HR: Hazard ratio
PDO: Patient derived organoids
PDX: Patient derived xenografts
IP injection: Intraperitoneal injection
OG: Oral gavage
RNA-Seq: RNA sequencing
DEGs: Differentially expressed genes
GO: Gene Ontology
GSEA: Gene Set Enrichment Analysis
NES: Normalized enrichment score
