# Supplementary material for: Targeting Myc-driven stress vulnerability in mutant KRAS colorectal cancer
Source: Mol Biomed. 2022 Mar 21;3:10. doi: 10.1186/s43556-022-00070-7 (PMC8934835; doi:10.1186/s43556-022-00070-7)

Targeting Myc driven-stress vulnerability in mutant *KRAS* colorectal cancer

Hang Ruan [1, 2], Brian J. Leibowitz [1, 2], Yingpeng Peng [1, 2], Lin Shen, [1,3,4], Lujia Chen [1, 5], Charlie Kuang [1, 6], Robert E. Schoen [1, 6, 7], Xinghua Lu [1, 5], Lin Zhang [1, 3], Jian Yu[1, 2 *]

[1]UPMC Hillman Cancer Center, 5117 Centre Ave., Pittsburgh, PA 15213, USA.

[2]Department of Pathology, University of Pittsburgh School of Medicine, Pittsburgh, PA 15213, USA

[3]Department of Pharmacology and Chemical Biology, University of Pittsburgh School of Medicine, Pittsburgh, PA 15213, USA

[4]Department of Oncology, Xiangya Hospital, Central South University, Changsha, Hunan, 410008, P.R. China

[5]Department of Medical Informatics, University of Pittsburgh School of Medicine, Pittsburgh, PA 15232, USA

[6]Department of Medicine, University of Pittsburgh School of Medicine, Pittsburgh, PA 15213, USA.

[7]Department of Epidemiology, University of Pittsburgh School of Public health Pittsburgh, PA 15213, USA.

[*]Correspondence:

Jian Yu, Hillman Cancer Center Research Pavilion, Suite 2.26h, 5117 Centre Ave, Pittsburgh, PA 15213. Email: yuj2@upmc.edu

Supplemental Materials and Methods

Cell culture, treatment, and transfection

For drug treatment, cells were plated in 12-well-plates at 20%-30% density 24 hours before treatment. DMSO (Sigma, St. Louis, MO, Cat#67-68-5) stocks of bortezomib (LC laboratories, Woburn, MA, Cat#179324-69-7) and everolimus (LC laboratories, Woburn, MA, Cat#159351-69-6) were prepared and diluted in cell culture media before adding to cells.

For transfection, Lipofectamine 2000 (Invitrogen, Cat#11668019) was used according to the manufacturer's instructions as [1,2]. In brief, the human eIF2AS51A construct was obtained from ADDGENE (#21808, Cambridge, MA). Cells were transfected with 0.4 µg of Plasmids/well in 12-well-plates for 4 hr. *GCN2* small-interfering RNA (siRNA) duplexes were synthesized by Dharmacon (Lafayette, CO, USA). Cells were transfected with 200 pmols of control scrambled or specific siRNA /well in 12-well plates for 4 hours. *MYC* siRNA and MYC expression plasmid have been described [2]. Transfected cells after 4 hours were incubation in medium containing 5% FBS for 20 hours, replated in 10% FBS normal growth medium for 24 hours before treatment. Details for siRNA sequence and expression plasmids are found in (Table S3).

Patient derived CRC organoids (PDOs)

The supplements included 1× penicillin/streptomycin (Invitrogen), 10 mM HEPES (Invitrogen), 2 mM GlutaMAX (Invitrogen), 1× B27 (Invitrogen), 1× N2 (Invitrogen), 1 mM N-Acetylcysteine (Sigma), 10 nM [leu-15]-Gastrin (Sigma), 10 mM nicotinamide (Sigma), 10 µM SB202190 (Sigma), 50 ng/mL recombinant murine EGF (Peprotech), and 0.5 µM A83-01 (Tocris Bioscience).

Organoids were passaged approximately 1:4 every 6–8 days with media change every 2–3 days as described [2,3]. Before treatment, organoids were digested into small clumps and seeded into 24-well or 96-well plates at appropriate density and cultured for 2 days. After treatment, cell viability was analyzed using the CellTiter-Glo® 3D Cell Viability Assay Kit (Promega, Cat#G9682) according to the manufacture's protocol. Organoids from 8 wells of each condition were pooled to prepare lysates and total RNA for western and qRT-PCR. Organoids from 16 wells of each condition were pooled, embedded in agarose gel, then fixed in 10% formalin and embedded in paraffin. H&E and immunostaining with p-eIF2a phosphorylation and cleaved-caspase 3 were performed on paraffin-embedded 5 μm sections as described [4].

Patient derived CRC xenografts (PDX)

PDX establishment. In brief, mice were housed in a sterile environment with micro isolator cages and allowed access to water and chow ad libitum. PDX1 was established from a KRASG13D and MS stable (MSS) (T4N0M1) tumor in the sigmoid colon of a 77-year-old male (4). PDX2 (ID #172845-121T) was obtained from NCI Patient-Deprived Models Repository (PDMR, https://pdmr.cancer.gov), and expanded in the lab. PDX2 was established from a KRASG12D, PI3KE545K, APCN1535Mfs*30, and MS stable adenocarcinoma in the colon of a deceased 43-year-old female after repeated therapies, including MK-2206, Selumetinib, Bevacizumab, FOLFOX, 5-Fluorouracil, Leucovorin, Indimitecan, FOLFIRI, Onalespib and Oxaliplatin.

We named first mouse passage implanted with the original human specimen as P0. ALL PDXs used were within 4 passages. For treatment experiments, we first expanded two pieces of frozen PDX stocks (~100 mm3) into NSG mice till approximately 1cm^3 (6-8 weeks). The expanded tumors were

harvested and divided into pieces (~100 mm3). Freshly cut pieces were immediately implanted into new NSG mice and allow to grow to palpable size in approximately 6-8 weeks prior to treatment.

Immunohistochemistry (IHC) and immunofluorescence (IF). Tumor tissues were dissected and fixed in 10% formalin and embedded in paraffin. Rehydrated sections were treated with 3% hydrogen peroxide (IHC only), followed by antigen retrieval for 10 min in boiling 0.1 M citrate buffer (pH 6.0) with 1 mM EDTA. p-eIF2a and cleaved-caspase 3 immunostaining was performed on 5 μm paraffin-embedded tumor sections as described [1,2]. Signals were detected by using AlexaFluor conjugated secondary antibody with nuclear counter staining by 4'6-Diamidino-2-phenylindole (DAPI).

Bioinformatics

Volcano plots are used to visualize the overall distribution of differentially expressed genes. The threshold of differential expression genes is both p-value<0.005 and log2(Fold Change) | > 1 (i.e.., 2-fold). Hierarchical clustering analysis is carried out of log10 (FPKM +1) of union differential expression genes, within all comparison groups [5]. Red denotes genes with high expression levels, and blue denotes genes with low expression levels. The color ranging from red to blue indicates log10(FPKM+1) value from the large to small.

Gene Ontology (GO) enrichment analysis of differentially expressed genes

Gene Ontology (GO) term analysis (Gene Ontology Resource) was applied to discover biological functions of the gene set of interest. The input is the genes of interest and the output is the enriched pathways with the significant p-value (≤0.05 or as specified). For shared or differentially expressed

genes among groups, Venn diagrams and gene lists were prepared using the function VennDiagram in R based on the gene list for different groups [6]. The display was limited to top 5 or 10 non-overlapping pathways with the most significant p-values.

Gene Set Enrichment Analysis (GSEA)

GSEA analysis [7] was performed with Molecular Signature Database (MSigDB. v7.4) using C2 and C5 datasets as indicated [8]. In brief, the input was the RNA-seq expression data of each pair (DEG no CutOFF). In the enrichment plot, the green curve is the enrichment score (ES) curve, which is the running sum of the weighted ES obtained from the GSEA software 4.1.0 (https://gsea-msigdb.org/ gsea/msigdb/index.jsp). The middle portion of the plot shows where the members of the gene set appear in the ranked list of genes. The bottom portion of the plot shows the value of the ranking metric as you move down the list of ranked genes. The normalized ES and the corresponding p-value are listed for all pairs as in Data deposit.

Supplemental Tables

Table S1. IC50s of translation-targeted agents in HCT 116 cells

Agent	Targets	IC50
Everolimus	mTOR/p-4EBP1 (multiple sites)	20-25 uM
Temsirolimus	mTOR/p-4EBP1 (multiple sites)	20-25 uM
Torin 1	mTOR/p-4EBP1 (multiple sites)	10-15 uM
Metformin	AMPK activator, mTOR/p-4EBP1	>100 uM
Sulindac	AMPK activator, mTOR/p-4EBP1	75-100 uM
eFT508	Mnk1/2, p-4E (S209)	30-40 uM
4EGI-1	eIF4E-G interaction	15-20 uM
4Ei-1	Cap, M7G Cap analog	> 500 uM
17AAG	eIF2a (43S PIC)	0.3-0.5 uM
Bortezomib	eIF2a (43S PIC)	15-20 nM
(Epi)silvestrol	eIF4A, helicase	8-10 nM

PIC, pre-initiation complex

Table S2. Cell line information

Cell Line	KRAS	BRAF	APC	CTNNB1	PIK3CA	PTEN	TP53
HCT116	G13D/+	WT	WT	S45del	H1047R	WT	WT
HCT116 (WT KRAS)	WT (+/-)	WT	WT	S45del	H1047R	WT	WT
HCT116 G13D?	G13(D/-)	WT	WT	S45del	H1047R	WT	WT
SW480	G12V	WT	Q1338*	WT	WT	WT	R273H, P309S
DLD-1	G13D	WT	R727M	WT	E545K,D549N	WT	S241F
DLD1 (WT KRAS)	WT (+/-)	WT	R727M	WT	E545K,D549N	WT	S241F
SW48	WT	WT	R2714C	S33Y	G914R	WT	WT
SW48/G12V (KI)	+/G12V	WT	R2714C	S33Y	G914R	WT	WT
RKO	WT	V600E	WT	WT	H1047R	WT	WT
DIFI	WT	WT	E1151*	WT	WT	WT	K132R
LS180	G12D	D211G	R1788C	S45F	H1047R	I67K	WT
Lim1215	WT	WT	WT	T41A,Q177P	WT	WT	WT

Table S3. chemicals, siRNA, and other key reagents

Name	Vendor	Catalog No.	Application/specifics
McCoy's 5A	Invitrogen	16600-082	CRC cell culture medium
DMSO	Sigma	67-68-5	solvent
Everolimus	LC Laboratories	E-4040	in vitro and in vivo
Bortezomib	LC Laboratories	B-1408	in vitro and in vivo
eIF2aS51 plasmid	Addgene	#21808	blocking p-eIF2a
GCN2 siRNA	Dharmacon	siGCN2-1383	CACCGTCAAGATTACGGACTA
CellTiter 96 AQueous One Solution Cell Proliferation Assay	Promega	G3580	Cell growth (MTS)
CellTiter-Glo® 3D Cell Viability Assay Kit	Promega	G9682	organoid growth
Crystal violet	Sigma	C0775-100G	cell visualization
PDO culture medium with supplements	various		PDO culture (see Ruan H et. al., Elife, 2020)

Table S4. Antibodies used in the study

ANTIGEN	VENDOR	CATALOG	APPLICATION	DILUTION
4EBP1	Cell Signaling	#9452S	IB	1/1000
4EBP1(S65/70)	Cell Signaling	#9451S	IB	1/1000
4EBP1(T37/46)	Cell Signaling	#2855	IB	1/1000
eIF2-alpha	Santa Cruz	SC-11386	IB	1/1000
eIF2 alpha (S51)	Cell Signaling	#3398	IB,IF	1/1000,1/100
Actin (Beta)	Sigma	A5441	IB	1/1000
Bim	Cell Signaling	#2819S	IB	1/1000
BiP (Grp78)	Cell Signaling	#3177	IB	1/1000
CREB-2 (ATF4)	Santa Cruz	SC-200	IB	1/1000
CHOP (L63F7)	Cell Signaling	#2895s	IB	1/1000
Caspase-3, cleaved	Cell Signaling	#9661	IB	1/1000
Caspase-8	Cell Signaling	#9746	IB	1/1000
Caspase-9	Cell Signaling	#9502	IB	1/1000
DR5	Prosci	2019	IB	1/1000
GCN2	Abcam	Ab137543	IB	1/1000
GCN2 (T899)	Abcam	Ab75836	IB,IF	1/500,1/100
KI67	DAKO	M7249	IF,IHC	1/100,1/100
Myc	Abcam	39688	IB	1/1000
NOXA	OP180	Sigma-Aldrich	IB	1/1000
PERK (T981)	Santa Cruz	SC-32577	IB	1/1000
PUMA	PMID: 12574499	#3795	IB	1/1000
S6 (S235/236)	Cell Signaling	#2211	IB,IF	1/1000,1/100
Goat anti-rabbit, Alexa594	Invitrogen	A11012	IF	1/200
Goat anti-mouse , Alexa488	Invitrogen	A11001	IF	1/200

Table S5. qRT-PCR primers used (Human)

Gene	Sequence
Actin-F	GACATTAAGGAGAAGCTGTGCTATGTT
Actin-R	GCCTAGAAGCATTTGCGGTGGACGA
ATF4-F	GTTCTCCAGCGACAAGGCTA
ATF4-R	GTGTCATCCAACGTGGTCAG
BIM-F	TAAGTTCTGAGTGTGACCGAGA
BIM-R	GCTCTGTCTGTAGGGAGGTAGG
CHOP-F	TGGAAATGAAGAGGAAGAATCAAAA
CHOP-R	CTTGGTGCAGATTCACCATTC
DR5-F	AAGACCCTTGTGCTCGTTGT
DR5-R	AGGTGGACACAATCCCTCTG
GADD34-F	ACTCCCCTAAAGGCCAGAAA

GADD34-R	GCTAAAGGTGGGTTCCTGAG
GADD45B-F	TACGAGTCGGCCAAGTTGATG
GADD45B-R	GGATGAGCGTGAAGTGGATTT
GCN2-F	TGGTAAACATCGGGCAAACTC
GCN2-R	GGACCCACTCATACAACAAGA
Myc-F	TCTTGACATTCTCCTCGGTGTCCGAGGACCT
Myc-R	TACCCTCTCAACGACAGCAGCTCGCCCAACTCCT
NOXA-F	ACCAAGCCGGATTTGCGATT
NOXA-R	ACTTGCACTTGTTCCTCGTGG
PUMA-F	GCCAGATTTGTGAGACAAGAGG
PUMA-R	CAGGCACCTAATTGGGCTC
XBP1-F	CCTTGTAGTTGAGAACCAGG
XBP1-R	GGGTCCAAGTTGTCCAGAATGC

Supplemental References

[1]He K, Zheng X, Li M, Zhang L, Yu J. mTOR inhibitors induce apoptosis in colon cancer cells via CHOP-dependent DR5 induction on 4E-BP1 dephosphorylation. Oncogene 2016;35(2):148-57. (In Eng). DOI: 10.1038/onc.2015.79.

[2]Ruan H, Li X, Xu X, Leibowitz BJ, Tong J, Chen L, et al. eIF4E S209 phosphorylation licenses myc- and stress-driven oncogenesis. Elife 2020;9. DOI: 10.7554/eLife.60151.

[3]Leibowitz BJ, Yang L, Wei L, Buchanan ME, Rachid M, Parise RA, et al. Targeting p53-dependent stem cell loss for intestinal chemoprotection. Sci Transl Med 2018;10(427). DOI: 10.1126/scitranslmed.aam7610.

[4]Wang X, Wei L, Cramer JM, Leibowitz BJ, Judge C, Epperly M, et al. Pharmacologically blocking p53-dependent apoptosis protects intestinal stem cells and mice from radiation. Sci Rep 2015;5:8566. DOI: 10.1038/srep08566.

[5]Montefusco DJ, Chen L, Matmati N, Lu S, Newcomb B, Cooper GF, et al. Distinct signaling roles of ceramide species in yeast revealed through systematic perturbation and systems biology analyses. Sci Signal 2013;6(299):rs14. DOI: 10.1126/scisignal.2004515.

[6]Chen H, Boutros PC. VennDiagram: a package for the generation of highly-customizable Venn and Euler diagrams in R. BMC Bioinformatics 2011;12:35. DOI: 10.1186/1471-2105-12-35.

[7]Subramanian A, Tamayo P, Mootha VK, Mukherjee S, Ebert BL, Gillette MA, et al. Gene set enrichment analysis: a knowledge-based approach for interpreting genome-wide expression profiles. Proc Natl Acad Sci U S A 2005;102(43):15545-50. DOI: 10.1073/pnas.0506580102.

[8]Leibowitz BJ, Zhao G, Wei L, Ruan H, Epperly M, Chen L, et al. Interferon b drives intestinal regeneration after radiation. Sci Adv 2021;7(41):eabi5253. DOI: 10.1126/sciadv.abi5253.

Supplemental figure legend

Figure S1. The BR combination induces apoptosis in mutant *KRAS* CRC cells. Indicated mutant CRCs were treated with vehicle (untreated, Un), Bortezomib (B), Everolimus (R), or their combination (BR, 5 nM and 10 μM) for 48 h. (a) Apoptosis was detected by flow cytometry. (b) Western blotting of indicated proteins at 24 h. The arrow indicates active form. Actin was used as the loading control.

Figure S2. The BR combination induces metabolic suppression and prolonged ISR. (a) Top 10 enriched pathways identified by GO in downregulated genes (1258) in HCT 116 cells at 24 h. (b) GSEA of DEGs in HCT 116 cells (BR *vs.* Un) (C5 dataset). Selected gene sets are shown with NES and corresponding *p*-value. (c) Cells were transfected with vector control (VC) or *EIF2AS51A* expression plasmid for 24 h, replated for 24 h, and treated by BR. qRT-PCR analysis of indicated genes at 24 h is visualized by heatmap. FC, fold change, values are normalized to vector transfected control cells (-BR as 1) for each cell line. (d) Apoptosis at 48 h was analyzed by nuclear fragmentation assay. Values are mean+s.d. (n = 3). **p<0.01 (Student's *t*-test, two tailed), VC *vs.* EIF2AS51A. (e) Apoptosis at 48 h was analyzed by flow cytometry and (f) quantitation of Annexin V+ cells.

Figure S3. BR induces ISR hyperactivation without affecting *MYC* mRNA. Indicated CRC cells were treated with vehicle (Un) or BR (5 nM and 10 μM). (a) qRT-PCR analysis of *MYC* mRNA at 24 h. (b) Western blotting of indicated proteins at different times. (c) qRT-PCR analysis of *GCN2* 48 h after siRNA transfection. (d) Cells were transfected with either scrambled (Ctrl) or

GCN2 siRNA for 24 h, replated for 24 h, and treated with BR for 48 h. Apoptosis at 48 h was determined using flow cytometry. a and c, values are mean+s.d. (n = 3). ***p<0.001 (Student's t-test, two tailed). siGCN2 *vs.* Ctrl.

Figure S4. BR induces mutant *KRAS*-selective ISR hyperactivation. CRC cells with mutant or wt*KRAS* were treated with vehicle (Un) or BR (5 nM and 10 μM). (a) Western blotting at 24 h. (B) qRT-PCR analysis at 24 h. Values are mean+s.d. (n = 3), normalized to untreated cells (1). ****p<0.0001 (Student's t-test, two tailed). Mutant *vs.* wt*KRAS* group. (c) Cluster analysis of BR (*vs.* Un) differential genes at 24 h. (d) Top 10 enriched pathways identified by GO in downregulated genes (1141), BR-treated WT *vs.* mutant *KRAS* HCT116 cells (fold change ≥ 2, p<0.005). (e) Top 10 enriched pathways identified by GO in upregulated genes (1176) in BR-treated WT *KRAS* HCT 116 cells. (f-g) Top BR DEGs (RNA-seq) in the isogenic pair visualized by heatmap. Log2FC, fold change, normalized to values in Un. mut *KRAS* (parental) HCT 116 cells (0).

Figure S5. BR induces ISR-associated killing of CRC PDOs.

(a) Representative images of PDOs after splitting 1: 3-5. Scale Bar = 100 μM. (b) qRT-PCR analysis at 24 h with values normalized to un (1). N=8 (pooled). Values are mean+s.d. (n = 3). *p<0.05, **p<0.01 (Student's t-test, two tailed). Un *vs.* BR. (c) Distribution of *KRAS* mutations in CRC subtypes (DFCI, Genetech, and MSKCC cohorts, n=1965) (cBioportal.org). (d) Correlation of mutant *KRAS* and OS in DFCI, Genentech, and MSKCC CRC cohorts (n= 978) (cBioportal.org). (e) Correlation of single gene expression and OS in the TCGA COAD cohort (n=270, All).

Figure S6. BR induces ISR hyperactivation and metabolic crisis in mutant *KRAS* CRC PDXs. PDX bearing NSG mice were treated with vehicle, or the combination of Everolimus (oral gavage; 10 mg/kg) and Bortezomib (i.p.; 0.5 mg/kg). (a) Treatment and analysis schedule. (b) Representative H&E staining and Ki-67 IHC of tumors on Day 17. Inserts are enlarged areas with arrows indicating well-formed glandular structures. Scale bar = 100 µM. (c) Quantification of Ki-67+ cells as in (b) in 3 randomly chosen 400x fields. (d) Body weight of mice. (e) qRT-PCR of indicated makers in tumors on day 4. The values were normalized to Un (1). (f) Top 10 enriched pathways identified by GO in downregulated genes (522). (g) Venn diagram of upregulated genes in HCT 116 and PDX1. (h) Top 5 enriched pathways identified by GO of shared genes from (g). c, e, values are mean+s.d. (n = 3). ***$p<0.001$, **$p<0.01$, *$p<0.05$ (Student's *t*-test, two tailed). Vehicle (Un) *vs.* BR.

Sfig.1, related to Fig 1

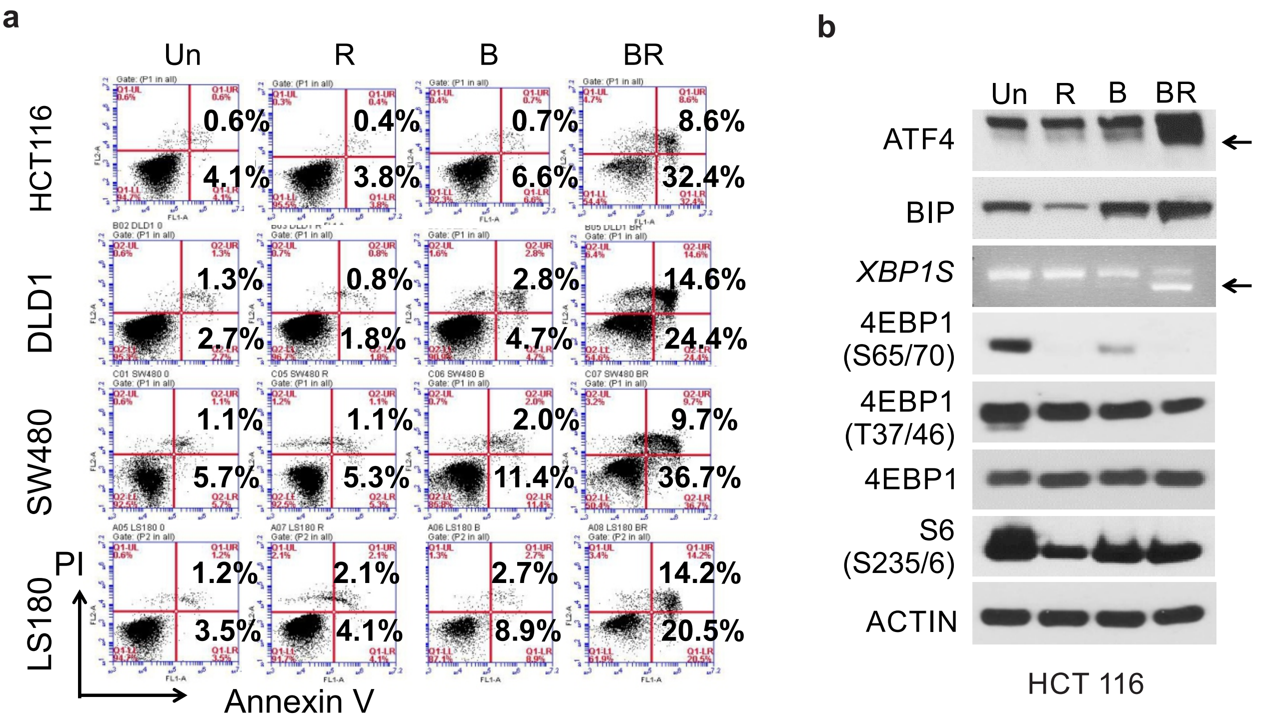

Sfig.2, related to Fig. 2

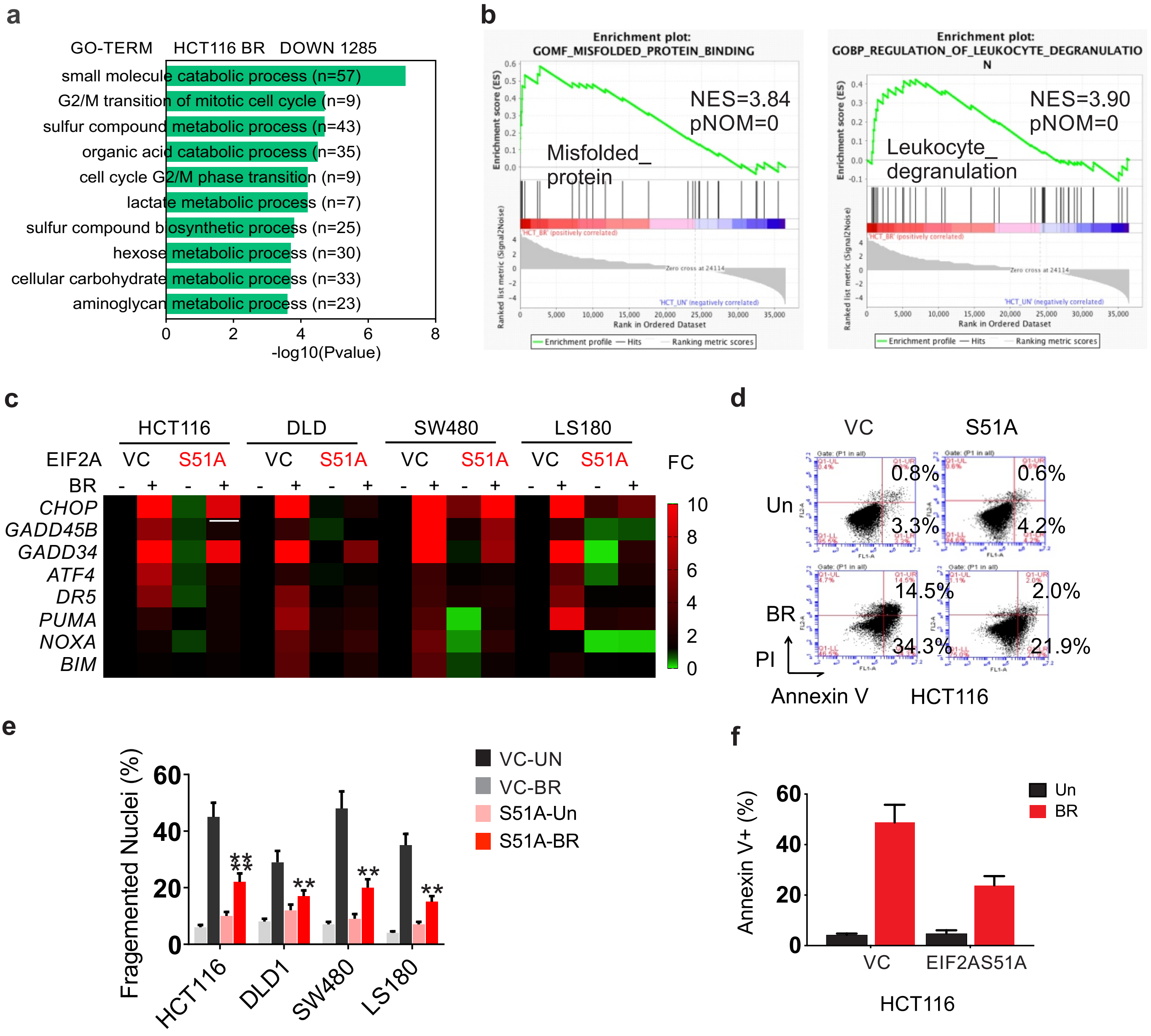

Sfig.3, related to Fig. 3

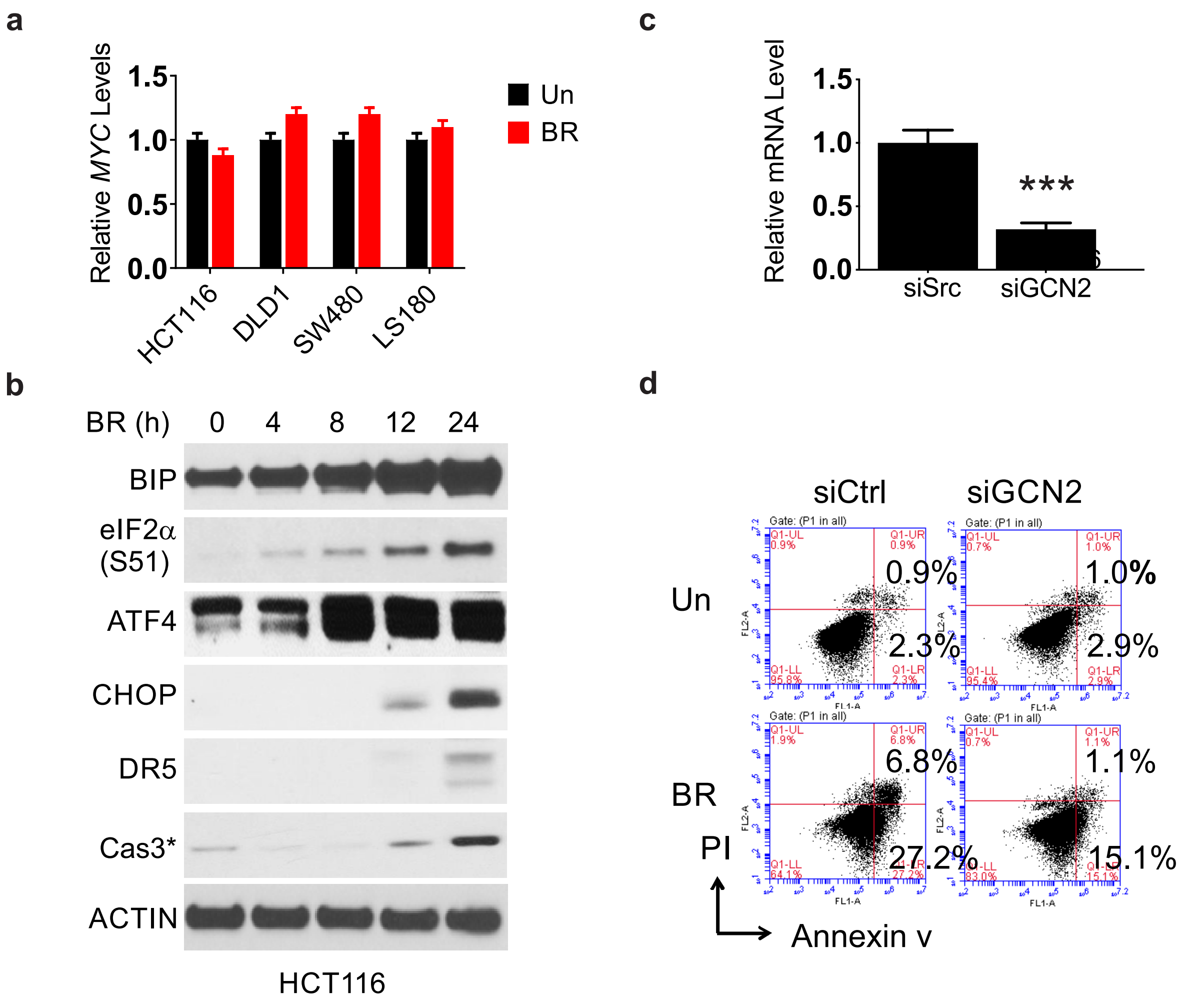

Sfig.4, related to Fig. 4

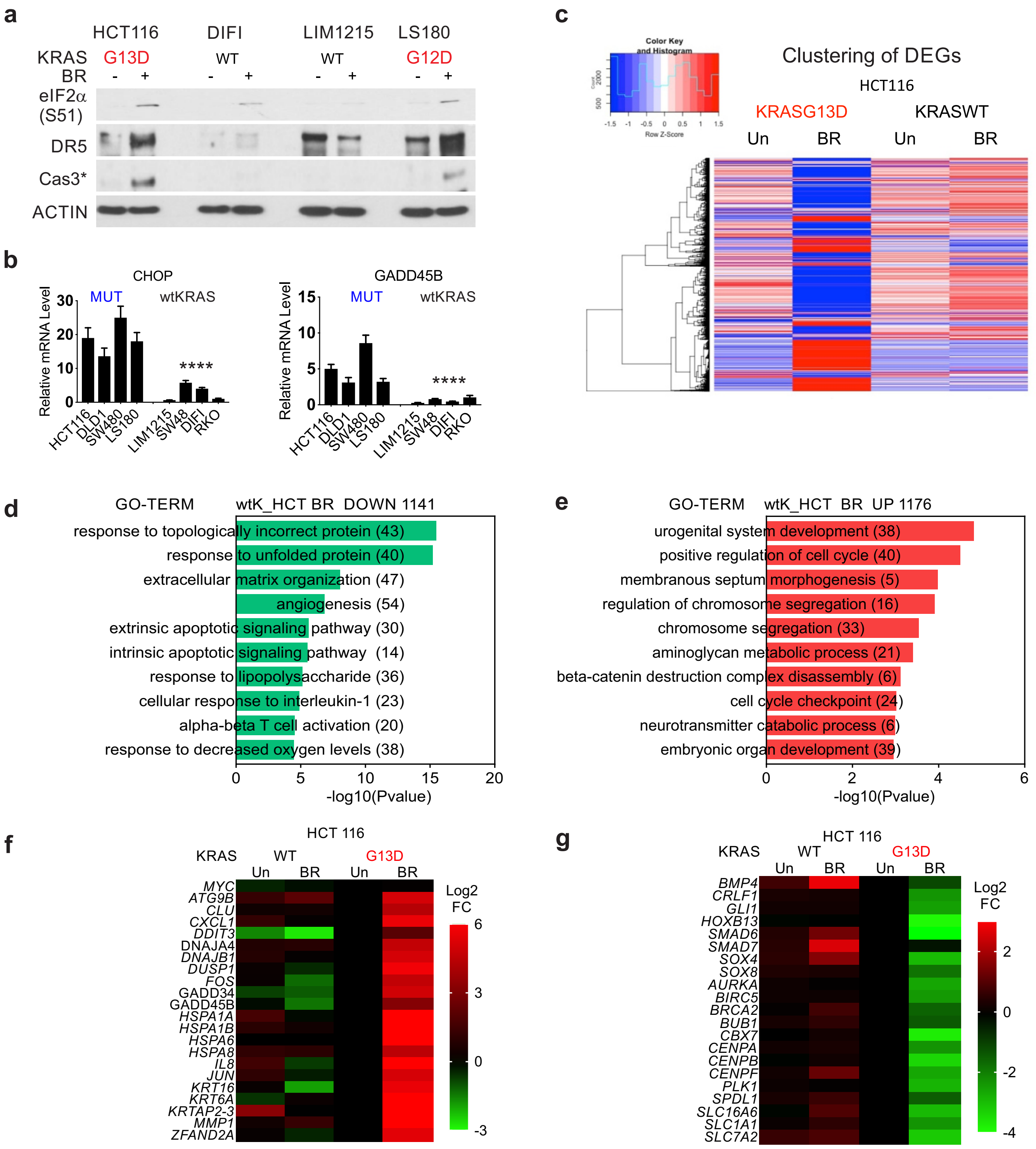

Sfig. 5, related to Fig.5

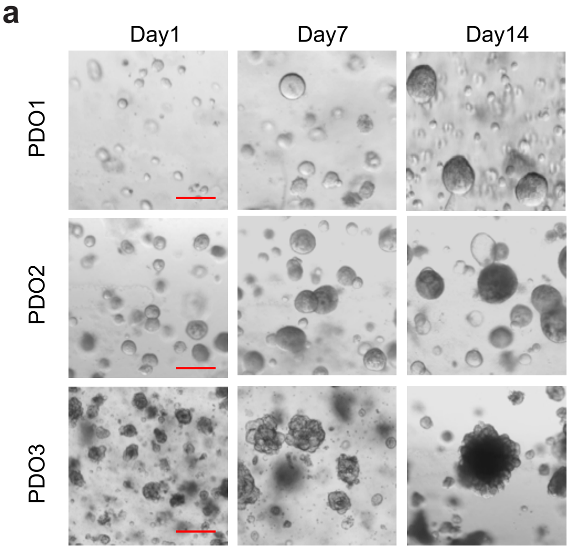

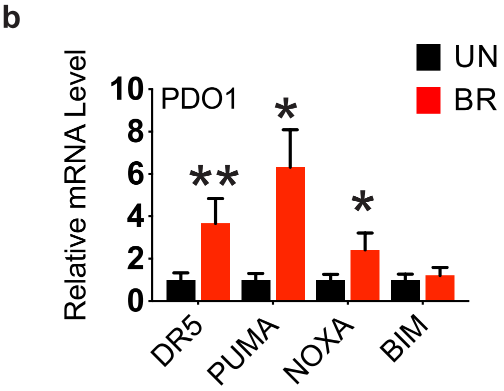

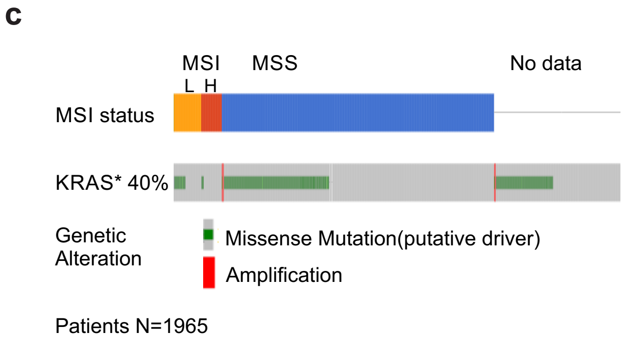

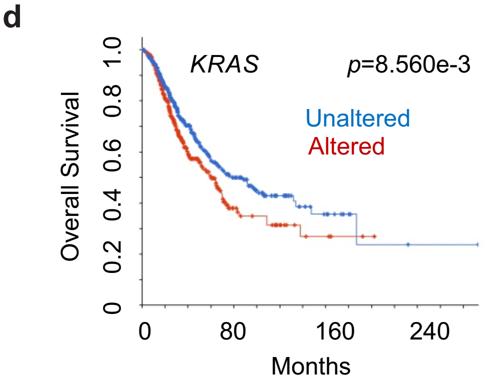

KRAS	Number of Cases	Median OS Months
Altered	447	59.93
Unaltered	531	78.73

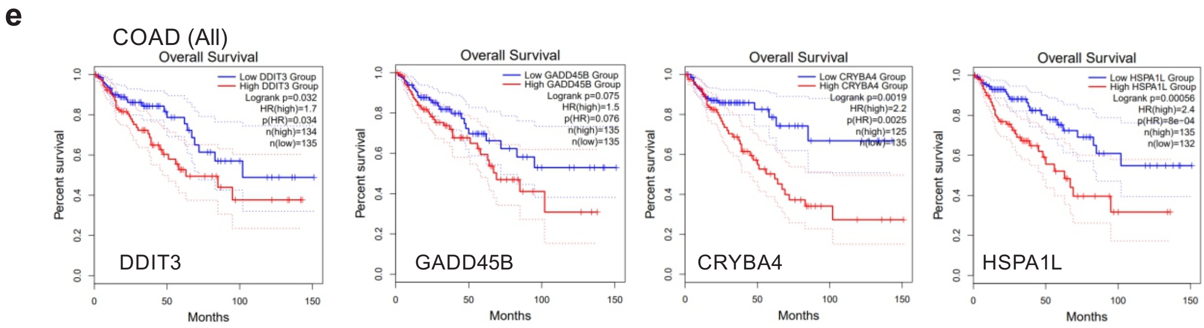

Sfig.6, related to Fig. 6

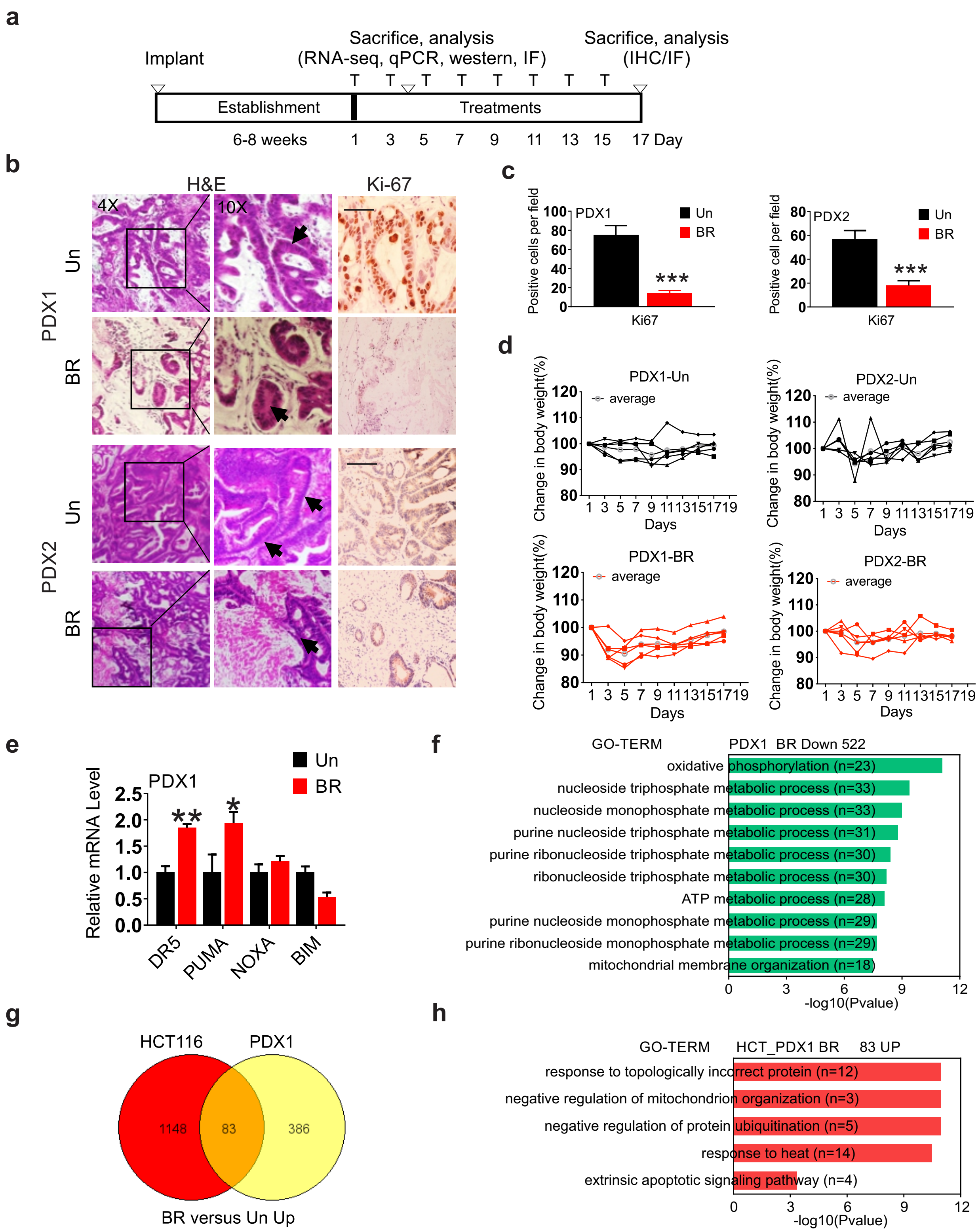

Supplement: Supplementary file 1 — Additional file 1: Supplementary Table S1. IC50s of translation-targeted agents in HCT 116 cells. Supplementary Table S2. Cell line information. Supplementary Table S3. chemicals, siRNA, and other key reagents. Supplementary Table S4. Antibodies used in the study. Supplementary Table S5. qRT-PCR primers used (Human). Supplementary Figure S1. The BR combination induces apoptosis in mutant KRAS CRC cells. Supplementary Figure S2. The BR combination induces metabolic suppression and prolonged ISR. Supplementary Figure S3. BR induces ISR hyperactivation without affecting MYC mRNA. Supplementary Figure S4. BR induces mutant KRAS-selective ISR hyperactivation. Supplementary Figure S5. BR induces ISR-associated killing of CRC PDOs. Supplementary Figure S6. BR induces ISR hyperactivation and metabolic crisis in mutant KRAS CRC PDXs. [file 43556_2022_70_MOESM1_ESM.pdf]
